# *Toxoplasma gondii* excreted‐secreted antigens suppress Foxp3 promoter activity via a SP1‐dependent mechanism

**DOI:** 10.1111/jcmm.15703

**Published:** 2020-07-29

**Authors:** Jinling Chen, Jingjing Wang, Xuyang Gao, Dandan Zhu, Liuting Chen, Yinong Duan

**Affiliations:** ^1^ Department of Pathogen Biology School of Medicine Nantong University Nantong China

**Keywords:** excreted‐secreted antigens, Foxp3 promoter, SP1, *Toxoplasma gondii*

## Abstract

*Toxoplasma gondii* excreted‐secreted antigens (ESA) could result in adverse outcomes of pregnancy including abortion, stillbirth, foetal infection or teratogenesis in mice during early stage of pregnancy. Defective generation or function of regulatory T cells (Tregs) may account for those adverse pregnancy outcomes. Forkhead box p3 (Foxp3), which is the key transcriptional factor of Tregs, modulates its development and maintains inhibitory function. We previously demonstrated that ESA inhibited Foxp3 expression by attenuating transforming growth factor β RII/Smad2/Smad3/Smad4 pathway. In this study, we propose to study the role of ESA on the activity of Foxp3 promoter and explore potential mechanisms. We demonstrated that ESA suppressed Foxp3 promoter activity using dual‐luciferase reporter assay. ESA functioned at −443/−96 region of Foxp3 promoter to suppress its activity using truncated fragments of Foxp3 promoter. Further analysis revealed that suppressive role of ESA on Foxp3 promoter activity is related to specificity protein 1 (SP1). Transfection of expression plasmid of pcDNA3.1‐SP1 could restore the down‐regulation of Foxp3 induced by ESA. In conclusion, this study provides a new mechanism by which ESA could inhibit the Foxp3 promoter activity via SP1.

## 1 INTRODUCTION

Toxoplasmosis, which is caused by *Toxoplasma gondii* (*T. gondii*), is regarded as the common zoonotic disease with a worldwide prevalence.^1^ *T. gondii* mainly infects humans through the ingestion of contaminated water or food with oocysts. Sometimes, it can also be transmitted to humans vertically during pregnancy. *T. gondii* infection in healthy individuals is regularly asymptomatic and self‐limited, while infection in pregnant individual may develop pathological conditions including abortion or congenital infection.^2^ Additionally, the severity of congenital toxoplasmosis is closely linked to infection timing during pregnancy.^3^ Maternal infection with *T. gondii* during early gestation could cause severe congenital toxoplasmosis; however, the infection during the late gestation invariably generates low infective rate of newborns.^4^, ^5^ Previous reports have demonstrated that immunopathological effects caused by *T. gondii* antigens are mainly responsible for the congenital toxoplasmosis.^6^ Consistent with those studies, our previous study suggested that ESA accounts for foetal absorption and teratogenesis during the early pregnancy stage in mice.^7^

A successful pregnancy relies on the maternal immune system that provides tolerance towards the semi‐allogeneic foetus without eliciting an immunopathological reaction.^8^ Regulatory T cells (Tregs) are an essential regulator on maintaining immune tolerance status of pregnancy.^9^ Our previous study indicated that Tregs number and function were down‐regulated in pregnant mice after ESA treatment. Foxp3, which belongs to the forkhead family and is a transcription factor encoded by X chromosome, can activate or repress its target genes to cooperatively regulate the development, function and homeostasis of Tregs.^10^, ^11^ In abortion‐prone mice, the decreased Foxp3 expression is associated with reduction of Tregs (CD4^+^CD25^+^) and may account for immune barrier disruption at foetal‐maternal interface.^12^ The key to initiate and regulate Foxp3 transcription is the conserved promoter sequence of Foxp3, which is located upstream of the transcriptional starting site (TSS).^13^ Our previous work demonstrated that ESA could inhibit Foxp3 expression via IL‐2Rγ/JAK3/Stats pathway^14^ and PI3K/AKT/mTOR signalling pathway.^15^ We propose to further study the effect of ESA on the activity of Foxp3 promoter and explore its potential mechanisms.

## 2 MATERIALS AND METHODS

### 2.1 Ethics approval

All in vivo experiments were approved under the Care and Use of Laboratory Animals (Ministry of Science and Technology of China, 2016). All the procedures were performed under the supervision of Nantong University of Animal Care Committee (number: 20170304‐001).

### 2.2 Preparation of excreted‐secreted antigens

1 × 10^5^ tachyzoites of *T. gondii* (Chinese I strain) infected female C57BL/6 mice every 3 days. ESA preparation was conducted according to our previous study.^6^ Briefly, tachyzoite was cultured for 3 hours in medium 1640 without 10% foetal bovine serum (FBS; Thermo Fisher Scientific). Collected cell supernatants were further concentrated with Amicon Ultra‐15 centrifugal filter devices by EMD Millipore. For the removal of endotoxin from ESA, Detoxi‐Gel Affinity Pak prepacked columns (Thermo Fisher Scientific) were utilized according to the manufacture's instruction.

### 2.3 Cell culture and treatment

EL4 lymphoma cell line (Cell Resource Center of Shanghai Institute of Life Science, Shanghai, China) was maintained in DMEM medium (Thermo Fisher Scientific) supplemented with 3.7 g/L sodium bicarbonate, 50 mmol/L 2‐mercaptoethanol, 10% FBS, 100 000 U/L penicillin and 100 mg/L streptomycin at 37°C in CO_2_ incubator. EL4 cells were stimulated with anti‐CD3 (precoated), anti‐CD28 (1 µg/mL) and TGF‐β (5 ng/mL), and then stimulated with 10 µg/mL ESA for 24 hours, while control groups were stimulated with 10 µg/mL non‐antigen‐specific stimulant ovalbumin (OVA).

### 2.4 Bioinformatics analysis of Foxp3 promoter and plasmids construction

A major mRNA start site was mapped and defined as position +1. The Foxp3 promoter sequence (−1711 bp to +179 bp) got from the National Center for Biotechnology Information (NCBI). Both PROMO network platform and JASPAR network tool software were used to analyse transcription factor binding sites.^16^ According to the instruction from the QIAamp^®^ DNA Micro Kit (Qiagen), isolated genomic DNA from EL4 cells was utilized to amplify Foxp3 promoter fragments as a template. And then, promoter fragments were cloned into pGL3‐Basic and pGL3‐Enhancer vector (Promega) to construct PB‐Foxp3 and PE‐Foxp3, respectively. Both truncated fragments and the SP1‐binding site mutant were originated from Foxp3 −1711/+179 promoter luciferase construction. Primers for generating the individual construction are listed in Table 1.

**Table 1:** Primers used in this study

Primer	Sequence (5′‐3′)	Purpose
Foxp3 F	CGAGCTCCACAAACATCAAGTTCCAGAGG	PB/PE‐Foxp3
Foxp3 A F	CGAGCTCGTGAGGGGAAGAAATCATAT	PE‐Foxp3 A
Foxp3 B F	CGAGCTCCACCAGACACAGCTCTGCTG	PE‐Foxp3 B
Foxp3 R	CCGCTCGAGGAGTTGCCTAAAGCTCC	Reporters^a^
Foxp3‐mut F	AAGAAAAAAAAACTACAAGAAATAAAAA GTAACCCTGCAAT	PE‐Foxp3 mut
Foxp3‐mut R	ATTGCAGGGTTACTTTTTATTTCTTGTAGT TTTTTTTTCTT	PE‐Foxp3 mut
SP1 1 F	GTCTCATAAGAAAAGAATAAACAAA	ChIP
SP1 1 R	AACTTTGCTTTTATACCGAGAA	ChIP
SP1 2 F	AAGAATAAACAAAGTAAGAGAGCAA	ChIP
SP1 2 R	TGCCACATTATCAAAAACAACT	ChIP

Abbreviations: F, Forward; R, Reverse.

^a^ Reporters: PB‐Foxp3, PE‐Foxp3, PE‐Foxp3 A, PE‐Foxp3 B.

### 2.5 Electroporation and reporter gene assays

EL4 cells were transfected by electroporation using BTX ECM830 Electroporator. Luciferase reporter vector containing 5 µg Foxp3 promoter fragments and 0.5 µg phRL‐TK with or without pcDNA3.1‐SP1 was added to 1 × 10^6^ EL4 cells resuspended in 250 µL electroporation solution. Then, cells were electroporated with settings of 200 V and 10 msec. After a 24‐hour culture, EL4 cells were exposed to ESA (10 µg/mL) or OVA (10 µg/mL) and cultured for further 24 hours. In some experiments, EL4 cells were treated with or without ESA after the transfection of PE‐Foxp3 A mut and pcDNA3.1‐SP1 together. Then, dual‐luciferase assay system (Promega) was performed to assay luciferase activity following the manufacturer's instructions.

### 2.6 Chromatin immunoprecipitation

Simple ChIP enzymatic chromatin immunoprecipitation kit (CST, Cell Signaling Technology) was utilized to perform chromatin immunoprecipitation (ChIP) following the manufacturer's instructions. In brief, for one chromatin preparation, 4 × 10^6^ cells were treated with 1% methanol‐free formaldehyde for 10 minutes. To block the reaction, glycine was added. The chromatin was harvested and fragmented using enzymatic digestion and sonication. Chromatin immunoprecipitation was performed with anti‐histone H3 (CST), anti‐SP1 (Santa Cruz Biotechnology) or anti‐IgG (CST). Anti‐histone H3 antibody and mouse IgG were utilized as positive and negative control, respectively. The immunoprecipitated chromatins were eluted with ChIP elution buffer and then treated with ribonuclease A and proteinase K. The DNA was amplified by two pairs of site‐specific primers (Table 1) by PCR.

### 2.7 Western blot

Proteins were extracted from cells treated with lysis buffer containing a cocktail of phosphatase and proteases inhibitors and then centrifuged at 800 *g* for 15 minutes at 4°C. Proteins were separated in SDS‐PAGE (10%), transferred to polyvinylidene fluoride (Life Technologies) membrane, blocked with 5% bovine serum albumin (BSA) for 1 hour at 20°C and immunoblotted with anti‐SP1 (Abcam), anti‐Foxp3, anti‐P65 (Santa Cruz Biotechnology) and GAPDH (CST) at 4°C overnight. Goat anti‐rabbit or horseradish peroxidase‐conjugated goat anti‐mouse IgG was secondary antibody, and then, immunoreactive proteins were revealed with enhanced chemiluminescence (Merck).

### 2.8 Statistical analysis

Statistical analyses were performed with Prism7 (Graphpad). For comparisons between only two groups, an unpaired two tailed *t* test was used to assess statistical significance. Statistical analyses for experiments with more than two groups were conducted with a one‐way ANOVA. *P* < .05 indicated statistical significance.

## 3 RESULTS

### 3.1 ESA suppressed the activity of Foxp3 promoter in EL4 cells

**Figure 1:**
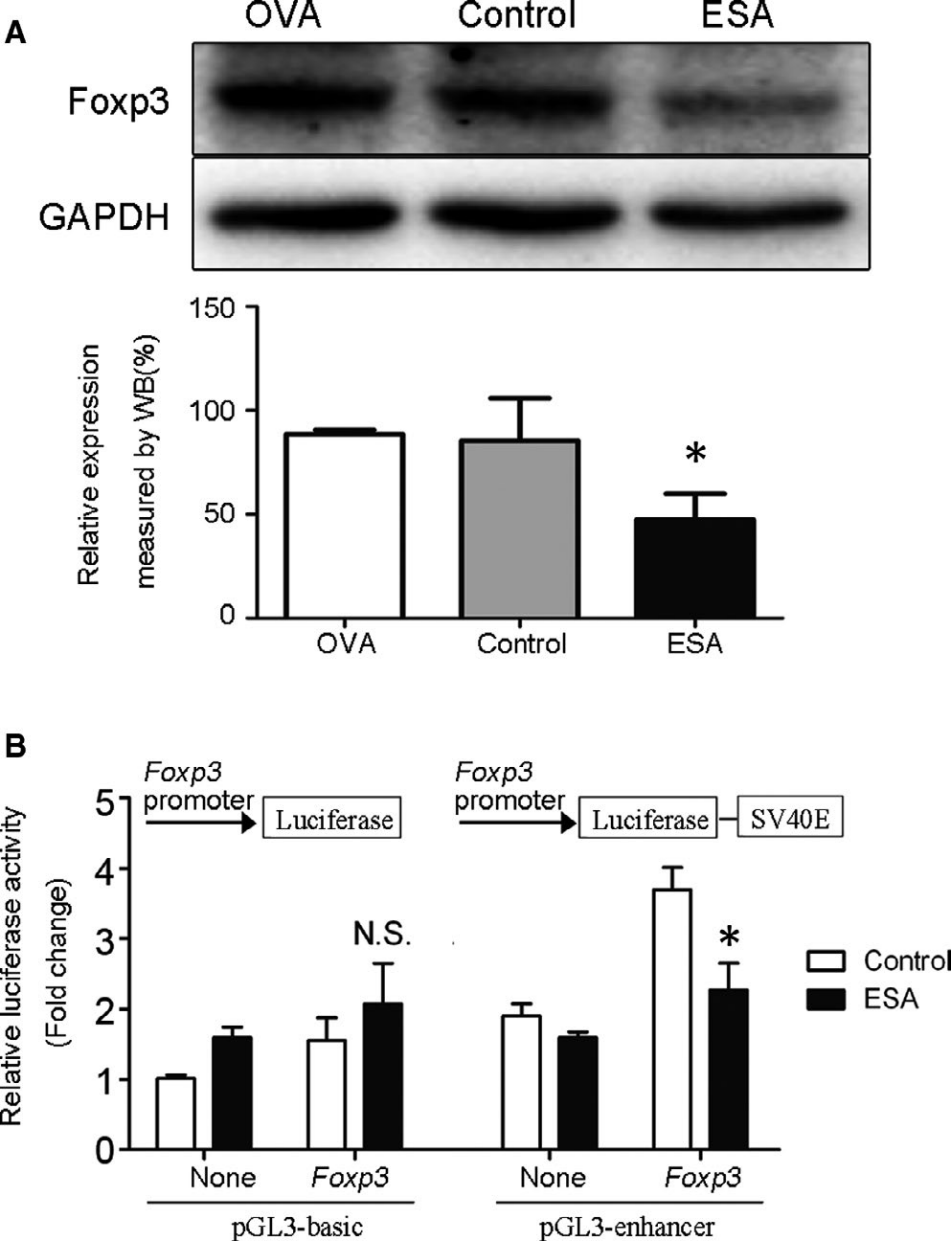
ESA inhibited the activation of Foxp3 promoter in EL4 cells. A, Foxp3 expression was measured by Western blot in EL4 cells treated with 10 µg/mL ESA or 10 µg/mL OVA. B, Fluorescence activity of Foxp3 promoter was detected by dual‐luciferase reporter assay in EL4 cells treated with ESA. NS: *P* >.05, no statistical significance. *P < .05

Foxp3, an essential molecular marker of Tregs, is a critical regulator in development, differentiation and maintenance of Tregs.^17^, ^18^, ^19^ To investigate the role of ESA on Foxp3 expression, EL4 cells was stimulated with ESA for 24 hours. Results indicated that the treatment of ESA could lead to the decrease in Foxp3 expression level in EL4 cells (Figure 1A). In our previous study, ESA exhibited an inhibitory effect on the mRNA level of Foxp3.^7^ Foxp3 promoter, located in the upstream of transcription start site, is a conserved sequence in the Foxp3 gene and is involved in initiating and regulating Foxp3 transcription.^13^ To further study the effect of ESA on Foxp3 at the gene level, EL4 cells were transfected with Foxp3 promoter luciferase reporter vectors by electroporation, and then treated with ESA for 24 hours. Unexpectedly, no activity of Foxp3 promoter was detected in Foxp3^+^ EL4 cells transfected with PB‐Foxp3 vector (Figure 1B). However, PE‐Foxp3 vector did enhance luciferase activity of Foxp3 promoter. It suggested that Foxp3 gene expression could be regulated through PE‐Foxp3 vector (Figure 1B). Therefore, we chose PE‐Foxp3 vector for subsequent experiments. A decrease in luciferase activity was observed when Foxp3 promoter was stimulated with ESA (Figure 1B). These results showed that ESA suppressed the activity of Foxp3 promoter in EL4 cells.

### 3.2 ESA functioned at −443/−96 region of Foxp3 promoter to inhibit its activity

**Figure 2:**
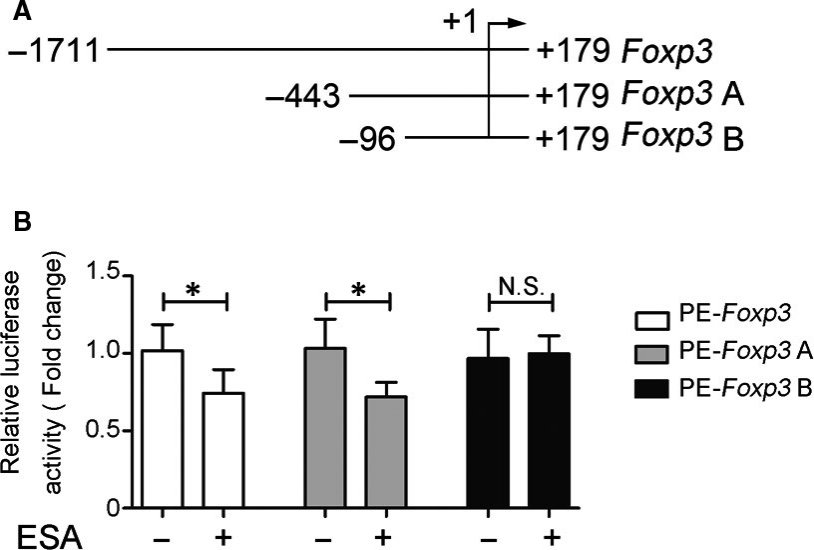
ESA functions at −443/−96 region of Foxp3 promoter to inhibit its activity. A, Diagram of luciferase reporter vectors having truncated fragments of Foxp3 promoter was showed. B, EL4 cells were treated with or without ESA. Dual‐luciferase reporter assay was utilized to detect fluorescence activities of PE‐Foxp3, PE‐Foxp3 A and PE‐Foxp3 B. NS: *P* > .05, no statistical significance. *P < .05

Aiming to narrow down the activity region, PE‐Foxp3 A and PE‐Foxp3 B, two luciferase reporter plasmids containing truncated fragments of Foxp3 promoter, were established (Figure 2A). To explore the underlining mechanism by which ESA inhibits Foxp3 promoter activity, PE‐Foxp3, PE‐Foxp3 A and PE‐Foxp3 B were transfected into EL4 cells, respectively. The transfected cells were stimulated with ESA for 24 hours. As shown in Figure 2B, Foxp3 promoter activity in ESA‐treated cells transfected with PE‐Foxp3 and PE‐Foxp3 A was significantly lower than that cells untreated with ESA. No significant difference was observed between ESA‐treated group and untreated group, which were transfected with PE‐Foxp3 B. It suggested that ESA might inhibit the activity of Foxp3 promoter in EL4 cells via binding to the −443/−96 region in Foxp3 promoter.

### 3.3 Transcription factor SP1 bound to the −443/−96 activity region of Foxp3 promoter

**Figure 3:**
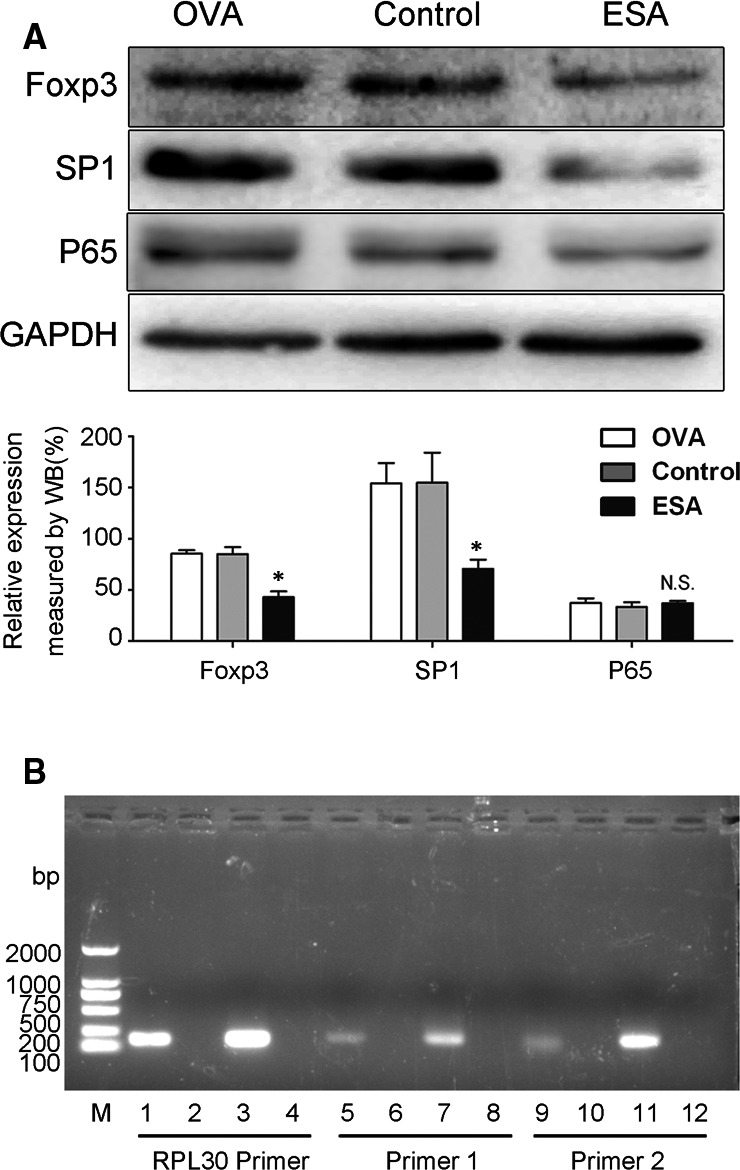
Transcription factor SP1 bound to Foxp3 promoter in −443/−96 activity region. A, The expression of SP1 and P65 after OVA or ESA treatment was assayed by Western blot. B, SP1 binding to Foxp3 promoter was confirmed by ChIP. M: DL2000 from SMOBIO. RPL30 Primer was utilized as positive control groups. Lane 1: anti‐Histone H3 group. Lanes 2, 6 and 10: normal IgG groups. Lanes 3, 7 and 11: input groups. Lanes 4, 8 and 12: water groups. Lanes 5 and 9: anti‐SP1 groups. NS: *P* > .05, no statistical significance. *P < .05

Both PROMO and JASPAR database were utilized to predict binding sites of transcription factor located in the −443/−96 activity region. Based on literature and bioinformatics analysis, specificity protein 1 (SP1) (−146/−155)^20^ and NF‐κB (−280/−289)^21^ transcription factor were selected candidates for transcriptional regulation of Foxp3 promoter. To explore whether both SP1 and NF‐κB are implicated in ESA regulation on the Foxp3 promoter activity, Western blot analysis was conducted to analyse SP1 and P65/NF‐κB expression after ESA treatment. We observed that ESA failed to down‐regulate P65/NF‐κB expression, but did diminish SP1 expression (Figure 3A). Therefore, we speculated that ESA might inhibit Foxp3 promoter activity in EL4 cells via a SP1‐dependent mechanism. To verify whether the SP1‐binding site was effective, primers were designed to perform ChIP analysis. Results indicated that SP1 bound to Foxp3 promoter at the −146/−155 activity region (Figure 3B). These data suggested that the inhibitory effect of ESA on Foxp3 promoter activity may be correlated to SP1 in EL4 cells.

### 3.4 ESA suppressed Foxp3 promoter activity via inhibiting SP1 expression

**Figure 4:**
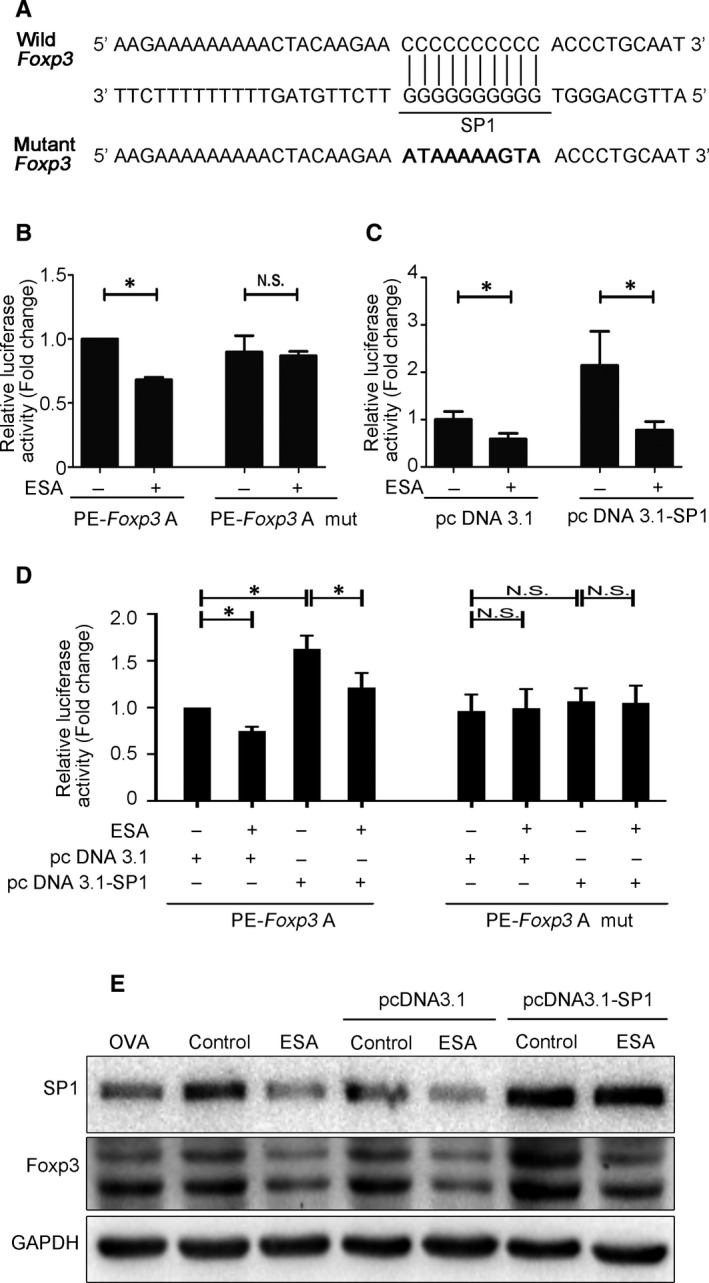
Role of SP1 on the regulation of Foxp3 promoter activity. A, Diagram of SP1‐binding site in the Foxp3 promoter region −146/−155 and SP1‐binding site mutant was showed. B, Dual‐luciferase reporter assay was used to detect the effect of ESA on the activity of PE‐Foxp3 and PE‐Foxp3 A mut in EL4 cells. C, The activity of PE‐Foxp3 was measured by dual‐luciferase reporter assay. EL4 cells were treated with or without ESA after the transfection of PE‐Foxp3 and pcDNA3.1‐SP1 or pcDNA3.1. D, Foxp3 promoter activity was measured by dual‐luciferase reporter assay. EL4 cells were treated with or without ESA after the transfection of PE‐Foxp3 A mut and pcDNA3.1‐SP1 together. E, The expression of Foxp3 and SP1 was assayed by Western blot after the transfection of pcDNA3.1‐SP1 or pcDNA3.1 vector into EL4 cells with or without ESA. NS: *P* > .05, no statistical significance. *P < .05

It has been demonstrated that SP1 is a critical regulator in Foxp3 gene transactivation through recruitment on Foxp3 promoter.^20^ We then attempt to investigate the effect of SP1 on Foxp3 promoter activity. To determine the effect of the SP1‐binding site in the region (−146/−155) of Foxp3 promoter, we mutated the binding site in a site‐directed manner (Figure 4A). Our results revealed ESA failed to inhibit the Foxp3 promoter activity in EL4 cells transfected with PE‐Foxp3 A mut (Figure 4B). To further characterize the role of SP1 on regulating Foxp3 expression, EL4 cells were transfected with Foxp3 promoter luciferase reporter vectors and pcDNA3.1‐SP1 expression vectors. As can be seen in Figure 4C, transfection with pcDNA3.1‐SP1 resulted in an obvious increase of Foxp3 promoter activity, while ESA treatment could suppress the activity of Foxp3 promoter. EL4 cells are transfected with PE‐Foxp3 A mut and pcDNA 3.1‐SP1 together. We found that ESA failed to affect luciferase activity of Foxp3 promoter. These data further revealed that ESA inhibited Foxp3 promoter activity in SP1‐dependent manner (Figure 4D). Moreover, the protein expression of SP1 and Foxp3 was significantly enhanced by pcDNA3.1‐SP1 in Figure 4E. ESA treatment caused the decrease in Foxp3 expression in EL4 cells transfected with pcDNA3.1‐SP1. These data indicated that ESA inhibited Foxp3 promoter activity via attenuating SP1 expression.

## 4 DISCUSSION

*Toxoplasma gondii* infection during pregnancy frequently causes abnormal pregnancy outcomes including spontaneous abortion, stillbirth, macro or microcephalus, hydrocephalus, and retinochoroiditis, though it is often self‐limited and asymptomatic in the mother.^22^ Maternal immune system providing tolerance towards the semi‐allogeneic foetus plays a vital role in a successful pregnancy.^8^ Tregs is key regulator in the development of immune‐tolerant environment.^9^ Studies have shown that the percentage and absolute number of circulating maternal Tregs enhance progressively during human pregnancy initiating from the first trimester.^23^ And then, levels reduce in the post‐natal period, though they are still higher than in pregnant control group. And it has proved that the increased levels of Tregs are linked with normal pregnancy,^23^, ^24^ whereas a reduced number of circulating Tregs is responsible for the immunological rejection of the foetus.^25^ Indeed, adoptive transfer with expanded Tregs isolated from pregnant mice could reduce abortion rate in abortion‐prone mice.^26^ Our previous studies have noted that ESA treatment in pregnant mice during the early pregnant stage resulted in spontaneous abortion, accompanied by decreased number and its function of Tregs.^7^ Accordingly, it seems that Tregs is critical to maintain maternal immune tolerance during pregnancy.

Foxp3, a lineage specification factor for Tregs plays an indispensable role on generating and maintaining regulatory T‐cell phenotypes.^27^ Foxp3 is expressed in Tregs precursors from thymus or periphery via the induction of TCR (T‐cell receptor) and cytokine signals. It has proved that ectopic Foxp3 expression in T cells could improve autoimmune symptoms in CD25^+^ T cell‐depleted mice.^18^, ^19^ Mutation of Foxp3 in mice leads to severe autoimmunity disease and multi‐organ infiltration owing to Tregs deficiency.^17^, ^18^ Foxp3 mutation in humans results in a similar autoimmune syndrome termed IPEX (immunodysregulation, polyendocrinopathy, enteropathy and X‐linked syndrome) with symptoms of insulin‐dependent diabetes, thyroiditis, enteropathy, infections, endocrinopathy and eczema.^28^, ^29^ In addition, Foxp3 ablation or attenuation in matured Tregs dysregulates Foxp3 target genes and compromised Tregs inhibitory function,^30^, ^31^ highlighting the role of Foxp3 on maintaining Tregs lineage identity and function. Previous work in our laboratory indicated that ESA could inhibit Foxp3 expression in vivo as well as in vitro. In this study, we further revealed that ESA could inhibit the Foxp3 promoter activity.

Given the importance of Foxp3 in Tregs generation and maintenance, it is necessary to explore the mechanisms of the regulation of Foxp3 gene. Brunkow et al have cloned 30.8 kb of Foxp3 genomic fragment, 12.5 kb of the 5′ flanking sequence and 2.8 kb of the 3′ flanking sequence. Non‐coding exon (named exon −2b) situating in the upstream of the first coding exon was discovered, and the region was highly consistent with the promoter region predicted by GENSCAN.^32^ Studies from Mantel et al further revealed that the Foxp3 promoter located 6.2 kb upstream of the Foxp3 translational start site. In addition, they identified several basal transcriptional elements, which are situated in the core promoter like TATA, GC and CAAT boxes.^20^ As a highly conserved sequence between humans, rats and mice, Foxp3 promoter activity is regulated by several transcription factors through the transcription factor binding sites within Foxp3 promoter.^20^, ^33^

Transcriptional regulation is accomplished through the binding of transcription factors to distinct promoter and enhancer elements. SP1 is one of the best characterized transcription factors in mammals,^34^, ^35^ which binds specifically to GC boxes and the identified motifs present in many promoters sequence.^36^ SP1 is omnipresently expressed in mammalian cells, and it is implicated in the regulation of many genes, such as housekeeping genes and inducible genes.^37^ SP1 regulates transforming growth factor‐β (TGF‐β) target genes in normal epithelial cells and epithelial tumour cells. Jungert revealed that Smad proteins and SP1 cooperatively regulate expression of a distinct set of TGF‐β target genes potentially involved in tumour progression, including MMP‐11, cyclin D1 and Smad7 in pancreatic cancer cells.^38^ In our previous study, we found that ESA negatively regulated Foxp3 expression via TGF‐βRII/Smad2/Smad3/Smad4 pathway.^7^ Thus, SP1 might be involved in the Smad2/Smad3/Smad4 pathway to modulate Foxp3 expression. SP1 protein contains two glutamine‐rich regions which are regarded as transactivation domains and a conserved zinc finger DNA‐binding domain.^39^, ^40^ Foxp3 promoter has identified a GC‐rich region via sequence scanning, and the region is bound by SP1.^20^ Additionally, the positive effect of SP1 on Foxp3 was observed, as the deficiency of SP1 blocked Foxp3 expression in CD4^+^ T cells.^41^

In conclusion, our current study reveals that ESA inhibits SP1 expression, thereby reducing its binding to Foxp3 promoter, ultimately resulting in Foxp3 loss. Our past and current studies provided potential mechanisms by which ESA regulated Foxp3 expression and therefore revealed an important pathway for targeted therapy of adverse pregnancy outcomes.

## CONFLICT OF INTEREST

All authors state that they have no conflicts of interest.

## AUTHOR CONTRIBUTION

**Jinling Chen:** Data curation (lead); Investigation (equal); Methodology (equal); Supervision (equal); Writing‐original draft (lead); Writing‐review & editing (equal). **Jingjing Wang:** Investigation (equal); Methodology (equal); Visualization (equal); Writing‐original draft (equal). **Xuyang Gao:** Investigation (equal); Methodology (equal); Project administration (equal). **Dandan Zhu:** Investigation (equal); Methodology (equal). **Liuting Chen:** Investigation (equal); Methodology (equal); Software (equal). **Yinong Duan:** Funding acquisition (equal); Supervision (supporting); Writing‐review & editing (supporting).

## Data Availability

The data that support the findings of this study are available from the corresponding author upon reasonable request.
